# Assessment of pulmonary fibrosis using weighted gene co-expression network analysis

**DOI:** 10.3389/ftox.2024.1465704

**Published:** 2024-10-24

**Authors:** Christina Drake, Walter Zobl, Sylvia E. Escher

**Affiliations:** Fraunhofer Institute for Toxicology and Experimental Medicine, Chemical Safety and Toxicology, Hannover, Germany

**Keywords:** WGCNA, bleomycin, lung fibrosis, diacetyl, DEG (differentially expressed gene) analysis

## Abstract

For many industrial chemicals toxicological data is sparse regarding several regulatory endpoints, so there is a high and often unmet demand for NAMs that allow for screening and prioritization of these chemicals. In this proof of concept case study we propose multi-gene biomarkers of compounds’ ability to induce lung fibrosis and demonstrate their application *in vitro*. For deriving these biomarkers we used weighted gene co-expression network analysis to reanalyze a study where the time-dependent pulmonary gene-expression in mice treated with bleomycin had been documented. We identified eight modules of 58 to 273 genes each which were particularly activated during the different phases (inflammatory; acute and late fibrotic) of the developing fibrosis. The modules’ relation to lung fibrosis was substantiated by comparison to known markers of lung fibrosis from DisGenet. Finally, we show the modules’ application as biomarkers of chemical inducers of lung fibrosis based on an *in vitro* study of four diketones. Clear differences could be found between the lung fibrosis inducing diketones and other compounds with regard to their tendency to induce dose-dependent increases of module activation as determined using a previously proposed differential activation score and the fraction of differentially expressed genes in the modules. Accordingly, this study highlights the potential use of composite biomarkers mechanistic screening for compound-induced lung fibrosis.

## Introduction

To date, risk assessment of industrial chemicals in the EU is regulated by the REACH legislation. Accordingly, compounds marketed at a very high tonnage have undergone full risk assessment, while for around 70% of the chemicals on the EU market hardly any toxicological data relating to hazards or exposure is available (European Environment Agency, 2019). Further, the mechanisms underlying adverse effects which have been detected *in vivo* remain often unknown for complex regulatory endpoints such as chronic toxicity after repeated exposure. There is therefore an unmet need to develop new approach methods that allow the testing and assessment of a wide range of chemicals, ideally with human relevant methods. New approach methodologies (NAMs), based on human *in vitro* and *in silico* models, offer this opportunity. In the context of the 3R paradigm shift, NAMs and integrated approaches to testing and assessment (IATAs) or defined approaches are under development in several European initiatives such as RISKHUNT3R (Pallocca et al., 2022) and the European Partnership for the Assessment of Risks from Chemicals (PARC) (Marx-Stoelting et al., 2023). Nevertheless, they are not yet implemented into regulatory decision making (ECHA, 2022; ECHA, 2023).

Read-across (RAx) is an often applied alternative method in which the data of one to several data rich compound (s) are used to predict the toxic properties of data poor target compounds (ECHA, 2022). RAx is also one area in which NAMs can already today be used to support the assessment of shared toxicodynamic and kinetic properties (ECHA, 2023; Escher et al., 2019; van der Stel et al., 2021). Read-across approaches offer some of the most regulatorily accepted opportunities to apply NAMs in a regulatory context (Ball et al., 2016; Schultz and Cronin, 2017). Omics (e.g., transcriptomics) read-outs provide useful information, because they can indicate adverse or adaptive response of a biological system to chemical stressors (Ganesan et al., 2021; European Commission: Directorate-General for Health and Food Safety, 2022; Johnson et al., 2022). ECHA (ECHA, 2023) as well as the recently published EFSA NAM roadmap report (Escher et al., 2022a) both highlight that regulatory acceptance of NAM methods requires a good understanding of their relevance, performance and remaining uncertainties and that case studies are an excellent tool to gain more confidence into them.

Case studies have shown the use of NAMs including (Q) SAR (Patlewicz et al., 2013), *in vitro* transcriptomics (Drake et al., 2023; Escher et al., 2022b; Vrijenhoek et al., 2022) and *in vivo* metabolomics (Kamp et al., 2024; van Ravenzwaay et al., 2016) to explore the mechanistic similarity of compounds and thereby add to the mechanistic evidence underpinning a potential read-across case.

The weighted gene co-expression network analysis (WGCNA) is a tool for transcriptome analysis which uses a network-based approach to evaluate genes’ joint activities. It has widely been applied to characterize gene involvement in disesases like idiopathic pulmonary fibrosis (Ghandikota, Sharma, Ediga, Madala and Jegga, 2022) or Alzheimer’s Disease (Liang et al., 2018). It has also been successfully applied to characterise the effects of chemical exposure on gene expression and to relate it to adverse outcomes (Callegaro et al., 2021). WGCNA enables the discovery of new biomarkers of adverse outcomes, i.e., both individual genes and sets of co-expressed genes that are associated with harmful chemical effects. It does not rely on pre-existing biological pathway information and is not limited or biased by this knowledge, which renders it a favorable alternative to enrichment analysis.

Biomarkers of pulmonary fibrosis have been identified using mainly transcriptomics data (including microarray, RNA-Seq and single-cell RNA-Seq) from lung tissue of humans found to suffer ideopathic pulmonary fibrosis (IPF). Data from IPF lungs describes the final state of lung fibrosis, but does not help us to understand the development of the disease (Vukmirovic and Kaminski, 2018). A WGCNA of IPF data identified modules for immune response, extracellular matrix or contractile fibres, developmental pathways of specific lung structures, cell division, DNA replication and DNA repair, cellular metabolic and catabolic processes, and surfactant metabolism (Mayr et al., 2021).

This study investigates a WGCNA of transcriptome data of the pulmonary fibrosis inducer bleomycin with the aim of identifying groups of genes with correlated expression patterns (modules). For this purpose, transcriptome data from an *in vivo* mouse study in which bleomycin was administered intratracheally for 35 days were used. The WGCNA analysis determined modules for different phases of disease progression starting from inflammation and progressing to acute and late fibrosis. The obtained eight modules were found to include 161 genes not previously associated with pulmonary fibrosis. These genes, as well as the entire identified modules represent novel biomarkers, which can be used to predict pulmonary fibrosis. To test this hypothesis, the modules were used to detect known fibrosis-causing compounds using *in vitro* derived transcriptome datasets.

## Materials und methods

Lung fibrosis related gene expression data: WGCNA analysis was applied to a publicly available *in vivo* pulmonary transcriptomics dataset for exposure to bleomycin, which is known to cause lung fibrosis (Peng et al., 2013). In this study, male C57BL6/J mice (n = 8 per group) were treated intratracheally with a dose of Bleomycin 2U/kg body weight. The whole lungs were extracted, homogenized and sampled at 7 time points on day 1, 2, 7, 14, 21, 28, 35 and after exposure. Based on histological findings, days 1 and 2 were classified as inflammentory phase, 7 and 14 as acute fibrotic phase and from day 21 onwards as late fibrotic phase. Whole transcriptome analysis was carried out using Affymetrix GeneChip Mouse Genome 430 2.0 arrays (GSE40151 (NCBI), Peng et al., 2013).

A recently published *in vitro* dataset on alpha-diketones ((Drake et al., 2023); biostudies dataset IDs S-TOXR1814, S-TOXR1825, S-TOXR1829 and S-TOXR1824; see https://www.ebi.ac.uk/biostudies/) was used for validation. In brief, (Drake et al., 2023), exposed primary bronchiolar epithelial cells (PBECs) to known and potential inducers of lung fibrosis for 1 hour using an air-liquid-interface. Test compounds included diacetyl and 2,3 pentanedione, which are known inducers of lung fibrosis (Zaccone et al., 2013), as well as the structural analogue 2,3-hexanedione, which is proposed by (Drake et al., 2023) to have the same mode of action. In addition, Drake et al. (2023) exposed the cells to negative and compound with another mode of action. Aceton served as negative control, as it did not induce any adverse effects in lungs in repeated dose toxicity studies (Bruckner and Peterson, 1981; Cavender, Casey, Salem, Swenberg and Gralla, 1983). Tunicamycin was tested as a compound with a different mode of action, which is disruption of protein folding (Wang et al., 2015). After 24 h, the cells were lysed and analysed with targeted transcriptome sequencing applying the S1500+ panel of the Templated Oligo-Sequencing (TempOSeq) technology (Drake et al., 2023). The S1500+ gene panel comprises 3,565 genes, which have been ensured to occur frequently in toxicological experiments and to cover known pathways (Mav et al., 2018).

### Differential gene expression analysis

Differential gene expression analysis of the bleomycin Affymetrix dataset was performed using the Genealyzer tool (Lietz, Saremi and Wiese, 2023), which is based on the R package limma (Ritchie et al., 2015). The quality control analysis did not show any gene or sample outliers with low read counts and/or batch effects in the Affymetrix dataset. The terms “transcript” and “gene” were used synonymously throughout this study.

The *in vitro* TempOSeq data was available in preprocessed form as counts per million (CPM) normalised read counts in text files (Drake et al., 2023) and analysed using DESeq2 (version 1.32.0; (Love, Huber and Anders, 2014)) in R. Genes were considered differentially expressed (DEG) if the adjusted p-value (padj) was <0.05 based on the Benjamini-Hochberg method and the absolute log2 foldchange was ≥1. (Bruckner and Peterson, 1981; Cavender et al., 1983; Drake et al., 2023; Wang et al., 2015; Zaccone et al., 2013).

### Weighted gene co-expression network analysis (WGCNA)

The analysis of the co-regulated genes was carried out specifically for gene expression in lung tissue using the R package WGCNA (Langfelder and Horvath, 2008). In a WGCNA, genes with similar expression patterns are grouped into modules (gensets). A module can be described using its so called eigengene and hubgene. The eigengene is a vector corresponding to the first principal (Sutherland et al., 2016) and therefore represents the largest changes in the expression profile. In contrast, the hub gene is an actual gene that corresponds most closely to the eigengene.

The samples were normalised with the count per million read (CPM) and log-transformed. The optimal soft threshold for the adjacency calculation was then determined graphically (power = 4). The cutreeDynamic function was used for tree pruning of the dendrograms of the hierarchical gene cluster, resulting in co-expression modules; correlated modules (r > 0.75) were then merged. The minimum module size was set to 30, as a pragmatic default value.

Modules were classified as relevant for the development of pulmonary fibrosis using a correlation score, for which the average of the absolute eigenvalues per phase (inflammation (days 1 and 2), acute fibrotic phase (days 7 and 14), late fibrotic phase (days 21 to 35) and controls) was determined. Also control samples achieved non-zero activity scores, therefore module selection was based on the ratio between the activities achieved in the three fibrotic phases and in controls, termed differential activity (DA). Modules with a DA greater than 2.5 were considered particularly active in the corresponding phase and were selected for further analyses.

The selected modules were then characterised using known biomarkers in the Disgent database (Piñero et al., 2015) and the hub genes. The hub gene is the gene that correlates most strongly with the eigengene.

For further characterisation of the modules, the R package gProfiler2 was used to perform a gene enrichment analysis of the genes in the modules. Enrichment of biological processes incuded in the Gene Ontology (GO) knowledgebase was determinedapplying Fisher exact test with adjustment for multiple testing through gProfiler2’s default method (padj <0.05) GO terms (biological processes) (Kolberg et al., 2020).

To apply the modules to the *in vitro* dataset as a proof-of-concept, mouse gene Ensembl IDs from the bleomycin dataset were mapped to human gene Ensembl IDs using Biomart. To compare the modules based on *in vivo* data with RNA data from *in vitro* experiments, the DEGs are compared with the genes in the module and an eigengene score is calculated per module and per DEG set of the *in vitro* experiment. For this purpose, a module score is calculated from the eigengene, which consists of the average absolute eigengene score across the co-expression modules (Sutherland et al., 2016). The module score thus describes how high the activation or repression of a module is on average. The activity scores were derived according to the method of (Callegaro et al., 2021).

## Results

### DEG analysis of the *in vivo* study on bleomycin

Peng et al. (2013) showed that bleomycin is a model substance which induces pulmonary fibrosis in mice. Based on histopathological findings, the authors described the progression of fibrosis in three phases after a single intratracheal exposure of 2U/kg body weight: an initial inflammatory phase (up to day 2), a subsequent acute fibrotic phase characterised by fibroblast infiltration and collagen formation (day 7 to 14), and a late fibrotic phase characterised by extracellular matrix remodelling (day 21 to 34). In the first 2 weeks after bleomycin exposure, the mice develop alveolar as well as interstitial fibrosis (Peng et al., 2013).

**FIGURE 1:**
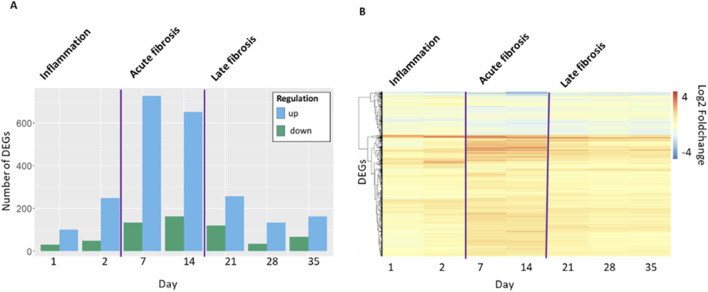
**(A)** *Number of differentially expressed genes (DEG) (p-adj = q <* 0.05, |log2FC| > 1*) in* mouse lung tissue *per timepoint in days after start of the exposure period. Up, increased expressing; down, decreased expression.* **(B)** *Hierarchical clustering based on log2 fold change for all genes that are DEG at least at one time point (N = 937).*

Gene expression at days 1 or 2 (inflammation); 7 or 14 (acute fibrosis) and 21 or 28 or 35 (late fibrosis) included a total of 937 of differentially expressed genes (DEGs). The number of DEGs (p-adj = q < 0.05, |log2FC| > 1) shows a time-dependent pattern (Figure 1). While only few DEGs (N = 274) are present in the inflammatory phase, the number of DEGs increases during the acute fibrotic phase to 720 up and 150 downregulated genes at day 7. These DEGs are characterized by particularly high fold changes. In the late fibrotic phase, the number of DEGs decreases again to less than 145 up or 61 downregulated genes (Figure 1).

### Weighted gene co-expression network analysis (WGCNA)

WGCNA was applied to the whole dataset including all measurements at all timepoints of exposed and control animals (Peng et al., 2013). Thereby, a total of 54 modules (MEs) of genes with highly positively or negatively correlated gene-expression were identified, including a total of 19,258 measured genes. ME size ranged from 33 to 5448 genes. For each module, an eigengene score was calculated for the expression of included genes in each of the histopathologically determined phases of the developing disease.

Eigengene scores (EG scores) are based on absolute values, higher values indicates higher activity. The EG scores per module in the inflammatory, acute fibrotic, and late fibrotic phases as well as in control animals shows highest activity levels in the inflammatory and acute fibrotic phase, while the activity decrease in the late fibrotic phase. Also control samples achieved non-zero activity scores, therefore ME selection employed DA, which is the ratio of the module to control EG scores (Table 1). Eight MEs have high DA (DA >2.5), namely modules 14, 20, 22, 28, 32, 37, 39, and 43. In total, these 8 MEs include 1039 mouse ensemble IDs of which 951 genes could be mapped to the human genome. The number of human genes in each of the individual modules ranged from 58 to 273. As expected, for all 8 selected MEs very little activity was found in control samples. DA values ranged across the eight selected modules from 1.02 to 4.76, 1.03 to 3.1 and 1.08 to 3.13 for the inflammatory, acute fibrotic and late fibrotic phases, respectively (Fehler! Verweisquelle konnte nicht gefunden werden).

**TABLE 1:** Characterisation of modules highly activated by bleomycine treatment *in vivo* (N = 8; differential activity >2.5). DA, differential activation.

Module ID	DA_inflamm_	DA_acute_	DA_late_	Hub gene	Hub gene short description	References
14	**4.76**	1.03	1.51	OAS1	Interferon-induced, dsRNA-activated antiviral enzyme which plays a critical role in cellular innate antiviral response. In addition, it may also play a role in other cellular processes such as apoptosis, cell growth, differentiation and gene regulation. (UniProt) past, human evidence	Chen, et al. (2017), Wickenhagen et al. (2021)
37	**2.54**	0.72	1.08	ZC3H18	No relevant information available.	
39	**2.70**	0.74	0.55	SPRR3	Inflammation (including inflammation in the lung) in human and mice	Zhu et al. (2021)
43	**2.66**	1.11	2.34	HPN	Serine protease that cleaves extracellular substrates, and contributes to the proteolytic processing of growth factors, such as HGF and MST1/HGFL. It plays a role in cell growth and maintenance of cell morphology as well as in the proteolytic processing of ACE2. Further, it mediates the proteolytic cleavage of urinary UMOD that is required for UMOD polymerization.	Ganesan et al. (2021), Herter et al. (2005), Heurich et al. (2014), Laidler et al. (1989), Torres-Rosado, et al. (1993)
32	1.02	**2.89**	1.50	TIPIN	Plays an important role in the control of DNA replication and the maintenance of replication fork stability. Important for cell survival after DNA damage or replication stress. May be specifically required for the ATR-CHEK1 pathway in the replication checkpoint induced by hydroxyurea or ultraviolet light. Forms a complex with TIMELESS and this complex regulates DNA replication processes under both normal and stress conditions, stabilizes replication forks and influences both CHEK1 phosphorylation and the intra-S phase checkpoint in response to genotoxic stress.	Baris et al. (2022), Cho et al. (2013), Unsal-Kacmaz et al. (2007), Yoshizawa-Sugata and Masai (2007)
20	2.37	1.43	**3.02**	CUTALP	Involved in resistance toward heavy metals, derived from mouse studies.	Adams et al. (2024)
22	**3.68**	**3.10**	**2.50**	LGI3	Suggested to relate to idiopatic pulmonary fibrosis (IPF), human evidence	Vastrad and Vastrad (2023)
28	**2.99**	2.02	**3.13**	VWA1	Extracellular matrix protein	Schupp et al. (2021)

DA values ≥2.5 are presented in bold font.

Modules 14, 37, 39, and 43 particularly differentially activated during the initial inflammatory phase. This finding is in line with the observation that the hub genes in ME 14 and 39 (OAS1 and SPRR3) are known to play a role in inflammation. No information is known about the ME 37s hub gene (ZC3H18). Module 43 is also relatively active in the late fibrotic phase. It’s hub gene (HPN) is involved in cleavage of extracellular substrates and maintenance of cell morphology, which is in line with the module’s association with fibrotic processes.

Module 32 and 20 are particularly differentially activated during the acute and late fibrotic phases, respectively. The ME32 hub gene (ENSMUSG00000032397,Tipin) is a check point gene, which is part of the DNA replication cycle (Gotter et al., 2007).

Modules 22 and 28 are differentially activated throughout the entire investigated time span. The hub gene of ME22 (LGI3) has been observed in patients suffering from ideopathic pulmonary fibrosis (IPF) and is particularly active across all three phases in this module. The hub gene of ME28 (VWA1) codes for a protein that is involved in the storage of collagen in the extracellular matrix, which is one of the main processes of fibrosis (Gebauer et al., 2016).

In order to get a broader and more robust impression of the association between highly activated modules and disease progression towards lung fibrosis, 924 known biomarkers of lung fibrosis from DisGenet (Piñero et al., 2015) were mapped to the genes included in the eight modules (Table 2). DisGenet is currently the largest database of human gene-disease associations.

**TABLE 2:** Characterisation of modules that are highly activated by bleomycine treatment *in vivo* (N = 8; differential activity >2.5). Number of human genes in the module (thereof included in the S1500+ gene panel) as well as lists of Ensembl gene IDs of genes considered to be biomarkers of lung fibrosis [according to DisGenet (Piñero et al., 2015)] and the top ten most significantly enriched (padj <0.05) GO terms (biological processes) per module. Bold Ensembl gene IDs are part of the S1500+ gene panel.

Module ID	# Genes: total (in S1500+)	Biomarker genes from DisGenet (bold - genes in the S1500+ panel)	Enrichment analysis: GO biological process
14	273 (42)	**ENSG00000184371** (CSF1) **ENSG00000115415** (STAT1) **ENSG00000172183** (ISG20) ENSG00000172156 (CCL11) ENSG00000178209 (KDELC2) ENSG00000138496 (PARP9) ENSG00000115267 (IFIH1) ENSG00000164808 (SPIDR) ENSG00000111537 (IFNG) ENSG00000147099 (HDAC8)	response to virus defense response to other organism response to biotic stimulus response to other organism response to external biotic stimulus biological process involved in interspecies interaction between organisms defense response to symbiont defense response to virus innate immune response defense response
37	86 (9)	ENSG00000134046 (MBD2) ENSG00000143543 (JTB) ENSG00000182220 (ATP6AP2) ENSG00000117298 (ECE1)	primary metabolic process organic substance metabolic process nitrogen compound metabolic process metabolic process organic substance biosynthetic process ncRNA metabolic process biosynthetic process cellular biosynthetic process cellular metabolic process organonitrogen compound metabolic process
39	83 (4)	No information	keratinization keratinocyte differentiation skin development epidermis development epidermal cell differentiation tissue development epithelial cell differentiation epithelium development animal organ development muscle system process
43	58 (12)	**ENSG00000132170** (PPARG) **ENSG00000167468** (GPX4) ENSG00000162733 (DDR2) ENSG00000187848 (P2RX2)	**-**
32	100 (17)	**ENSG00000100311** (PDGFB) **ENSG00000130816** (DNMT1) **ENSG00000145147** (SLIT2) ENSG00000087494 (PTHLH)	regulation of nitrogen compound metabolic process regulation of primary metabolic process vascular associated smooth muscle cell differentiation regulation of vascular associated smooth muscle cell differentiation regulation of macromolecule metabolic process
20	167 (29)	**ENSG00000168036** (CTNNB1) **ENSG00000160179** (ABCG1) **ENSG00000169710** (FASN) **ENSG00000164111** (ANXA5) **ENSG00000135318** (NT5E) **ENSG00000150782** (IL18) **ENSG00000185499** (MUC1) ENSG00000004142 (POLDIP2) ENSG00000164690 (SHH) ENSG00000140092 (FBLN5) ENSG00000164251 (F2RL1) ENSG00000172270 (BSG) ENSG00000137648 (TMPRSS4) ENSG00000085063 (CD59)	positive regulation of biological process regulation of cellular localization macromolecule localization organic substance transport positive regulation of cellular process regulation of localization regulation of protein localization biological regulation nitrogen compound transport establishment of protein localization
22	149 (25)	**ENSG00000185303** (SFTPA2) **ENSG00000122852** (SFTPA1) **ENSG00000253729** (PRKDC) **ENSG00000245848** (CEBPA) ENSG00000204305 (AGER) ENSG00000167972 (ABCA3) ENSG00000113578 (FGF1) ENSG00000143507 (DUSP10)	cell morphogenesis organonitrogen compound metabolic process neuron projection morphogenesis plasma membrane bounded cell projection morphogenesis cell projection morphogenesis cellular catabolic process cell morphogenesis involved in neuron differentiation system development multicellular organism development establishment of localization
28	123 (19)	**ENSG00000119699** (TGFB3) ENSG00000111087 (GLI1) ENSG00000213694 (S1PR3)	positive regulation of myeloid dendritic cell chemotaxis mesangial cell-matrix adhesion regulation of myeloid dendritic cell chemotaxis negative regulation of cellular extravasation negative regulation of leukocyte tethering or rolling negative regulation of leukocyte adhesion to vascular endothelial cell myeloid dendritic cell chemotaxis negative regulation of dendritic cell apoptotic process regulation of multicellular organismal development regulation of cellular extravasation

On top of that, enriched biological processes (GO; padj <0.05) were determined (Table 2). Statistically significantly enriched biological processes included inflammation-related processes such as innate immune response, regulation of cellular extravasation, as well as fibrogenesis-related processes such as epithelial development, cellular catabolism, cell differentiatiation and cell (projection) morphogenesis, establishment of (macromolecule) localization and system development (Dörr and Herrmann, 2003).

Module 20, which shows particularly high DA during the late phase of lung fibrosis development, comprises the highest number of known biomarkers for lung fibrosis, with 14 of a total of 167 genes. The number of biomarkers included in the remaining selected modules ranged from 14 to 3, except for module 39, which does not include any known biomarker of lung fibrosis listed in DisGenet.

### Application of the eight biomarker modules

The eight modules were tested for their applicability as potential biomarkers to identify fibrosis inducing chemicals. For this purpose, a recently published dataset (Drake et al., 2023) on human primary bronchiolar epithelial cells (PBECs) exposed to known induncers of lung fibrosis was used.

**FIGURE 2:**
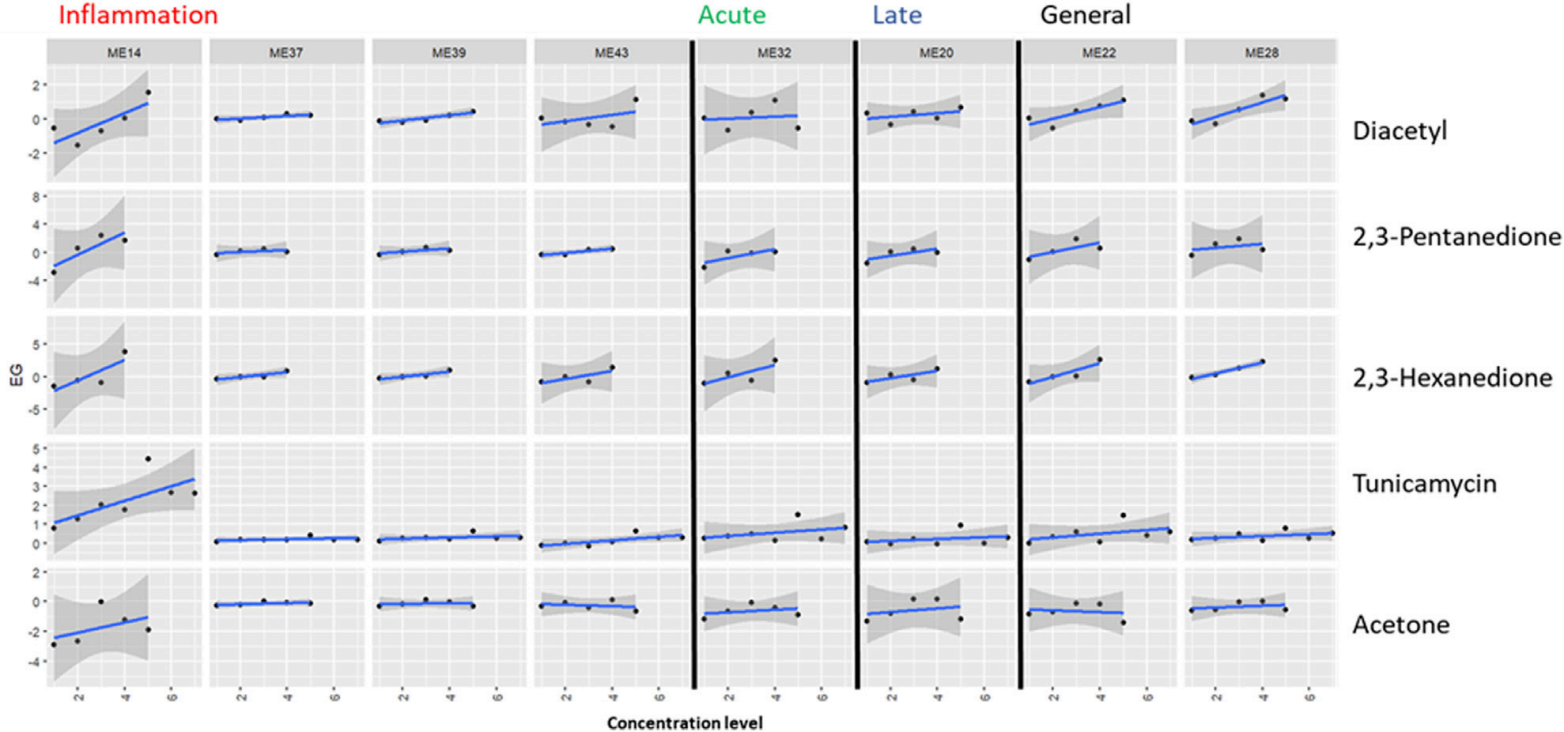
Application of the proposed biomarker modules (ME) for compound induced lung-fibrosis to an external dataset. Module activation by three lung-fiborisis inducing compounds (diacetyl, 2,3-pentanedione, 2,3-hexanedione) and two compounds with other modes of action is expressed as the fraction of the number of S1500+ genes in the module, which have been found to be differentially expressed (DEG) in human primary bronchial epithelial cells after exposure for 24 h at 4 to 7 subcytotoxic concentration levels by (Drake et al., 2023). Module annotations point to the histopathologically determined phase of bleomycin-induced development of fibrosis (inflammation, acute fibrotic, late fibrotic, or several (general)) which a given module was found to be most differentially activated in. Dashed lines indicate the level where 20% of measured genes are differentially expressed.

**FIGURE 3:**
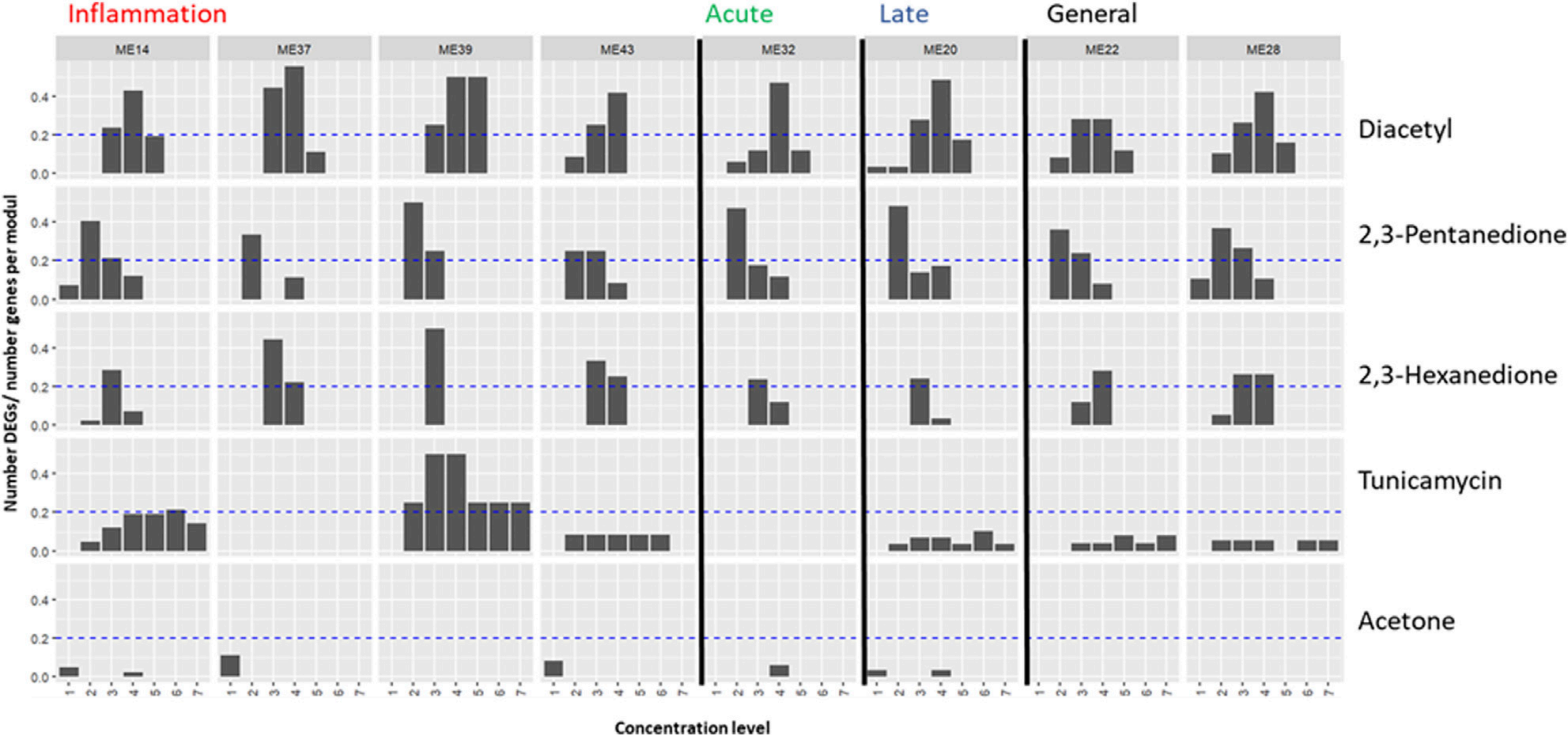
Application of the proposed biomarker modules (ME) for compound induced lung-fibrosis to an external dataset. Module activation by three lung-fiborisis inducing compounds (diacetyl, 2,3-pentanedione, 2,3-hexanedione) and two compounds with other modes of action is expressed as eigengene scores based on *in vitro* transcriptomics read-outs after exposure for 24 h at 4 to 7 subcytotoxic concentration levels presented by (Drake et al., 2023). Blue lines and grey shapes depict linear regression models and the associated 95% confidence intervals, respectively. Module annotations point to the histopathologically determined phase of bleomycin-induced development of fibrosis (inflammation, acute fibrotic, late fibrotic, or several (general)) which a given module was found to be most differentially activated in.

Since Drake et al. (2023) measured a small panel of genes, this proof-of-concept analysis could only take into account 42 to 4 genes per module (see Table 2). Based on these genes, module activation was assessed in two ways. The number of DEGs in a given module as determined by Drake et al. (2023) at each test compound concentration was shown as a fraction of the total number of measured genes in the module (Figure 2). Second, differentially expressed genes at more than one test compound concentration were listed in Table 3. Additionally, dose-responsiveness of eigengene scores was assessed per module and test compound (Figure 3).

**TABLE 3:** Ensembl gene IDs of DEGs in the proposed eight biomarker modules determined in an external *in vitro* validation dataset for three known lung-fibrosis inducing compounds (Drake et al., 2023). DEGs are listed per module, when differentially expressed in at least two tesed concentrations per compound. Bold ensemble gene IDs indicate DEGs found in more than one of the compounds.

Module ID	Diacetyl	2,3 Hexandione	2,3-Pentandione
ME14	**ENSG00000185507** (IRF7) ENSG00000112249 (ASCC3)		**ENSG00000185507 (IRF7)** ENSG00000132530 (XAF1) ENSG00000137628 (DDX60) ENSG00000204209 (DAXX) ENSG00000134326 (CMPK2)
ME37	ENSG00000160208 (RRP1B)		
ME39	ENSG00000170477 (KRT4)		ENSG00000181458 (TMEM45A)
ME43			
ME32	ENSG00000139687 (RB1)		ENSG00000116761 (CTH)
ME20	ENSG00000070731 (ST6GALNAC2) ENSG00000163686 (ABHD6)		ENSG00000135480 (KRT7)
ME22	**ENSG00000168621 (GDNF)** ENSG00000138085 (ATRAID)		**ENSG00000168621 (GDNF)** ENSG00000245848 (CEBPA)
ME28		**ENSG00000105290 (APLP1)**	**ENSG00000105290 (APLP1)** ENSG00000006652 (IFRD1) ENSG00000189056 (RELN)

Genes that are differentially expressed in more than one substance are labelled in bold.

Diacetyl induced pronounced differential expression in all of the 8 MEs. More than 20% of measured genes were differentially expressed at two to three diactyl concentration levels for the modules strongest associated with the inflammatory phase of bleomycin-induced lung-fibrosis, one concentration level for module 32 (association with the acute fibrotic phase), and at two concentration levels for module 20 (late fibrotic phase association) and modules 22 and 28 (generally high differential activation by bleomycin across phases, Figure 2). However, fractions of differentially expressed genes were generally not concentration responsive. In contrast, EG scores showed a concentration-responsive tendency for ME 14 (inflammatory phase) as well as ME 22 and ME 28 (high DA across phases, Figure 3). Further, EG scores for ME 43 (inflammatory phase) and ME 32 (acute fibrotic phase) indicated a concentration-dependent response with only the highest tested concentration.

2,3-pentanedione and 2,3-hexanedione, which have been shown to trigger lung fibrosis based on a similar mechanism of action as diacetyl, induced differential expression of >20% of the measured genes at least at one concentration level for all modules but–again–without concentration responsiveness comparable to diacetyl (Figure 2). Cells treated with 2,3-pentanedione and 2,3-hexanedione showed concentration-dependent response in the EG scores of modules 14, 32 and 22 and of modules 14, 43, 32, 22 and 28, respectively.

Tunicamycin, a substance with a known different mode of action causing protein missfolding activated two modules associated with the inflammatory phase (14 and 39), but none of the modules associated with the fibrotic phases (Figure 2). A dose responsive tendency of the EG score can only be found for module 14 (Figure 3).

Acetone, which had been found not to be toxic when inhaled, induced little differential expression overall and it was well below the threshold of 20% of measured genes for all modules, at all tested concentrations (Figure 2). EG scores showed some tendency to increase concentration-dependently for module 14, but not for any other or the selected modules (Figure 3).

Interestingly, IRF7 (interferon regulatory factor 7, ENSG00000185507; part of module 14) was differentially expressed after treatment with the known fibrosis inducers diacetyl and 2,3-pentanedione at two test compound concentrations each (Table 3). This is a known inflammatory gene, which is also discussed as a link between inflammation and fibrosis (Wu et al., 2019). Further genes that were differentially expressed at least at two test concentrations of more than one of the inducers of lung-fibrosis include GNDF (ENSG00000168621, module 22) and ALP1 (ENSG00000105290, module 28). GNDF plays a role in various inflammatory processes, including lung inflammation (Morel, Domingues et al., 2020). ALP1 belongs to the APP gene family, which plays a role in cell growth, among other things, as its protein products can bind particularly well to collagen (Beher et al., 1996) (Table 3).

## Discussion

To date transcriptome data are seldomly used in regulatory risk assessment of chemicals and one challenge is the missing link of chemically induced changes at the molecular level to their adverse outcomes (AOs) (Brockmeier et al., 2017). A frequently applied technology for interpretation of transcriptome data is a gene set enrichment analysis, which applies a defined set of genes like a molecular signaling pathway for data interpretation (Dean et al., 2017). This approach is, however, dependent on prior knowledge regarding pathways leading to adverse otucomes and it can be assumed, that neither the pathway databases nor the current AOPs are covering all relevant adverse outcomes for endpoints such as systemic toxicity after repeated long-term exposure. Therefore, case studies are needed to complement the annotation of genes to adverse outcomes and to gain confidence into their application.

Lung fibrosis is a frequently observed adverse outcome in humans and typically develops over a long period of time e.g. after exposure at the work place (Zaccone et al., 2013), to environmental pollutants (Harari et al., 2020) or most frequently to smoking (Morse and Rosas, 2014). Therefore, human samples of fibrotic lungs usually represent the final stage of this process (Prasse et al., 2019). In order to follow the different phases from early inflammatory responses to acute and late fibrosis, the present study is based on a mouse dataset, in which the known fibrosis inducer bleomycin has been applied. While interspecies differences and the incomplete mapping of genes from mouse to humans are a disadvantage of this approach, the evaluation of early changes are a clear advantage for data interpretation.

A number of *in vitro* studies have demonstrated that short-term exposure, like 4 h or 24 h, of cell models, such as bronchial epithelial cells, results in transcriptional changes that are indicative of the subsequent development of pulmonary fibrosis (Saarimäki et al., 2020; Xu et al., 2019). As the majority of cellular models are unable to capture the whole development of pulmonary fibrosis, the identification of early biomarkers is of particular importance for their interpretation and inference of pulmonary fibrosis.

Most DEGs in the mouse dataset utilized in this study belong to the acute fibrotic phase. In this phase, the fraction of upregulated DEGs is particularly high assumably indicating the induction of a large number of cellular processes related to the remodelling of the pulmonary tissue.

The WGCNA detected 54 modules of genes with correlated expression patterns indicating an involvement in the same cellular processes. Eight of these 54 modules constitute potential composite biomarkers showing considerably increased activity in the inflammatory or fibrotic phases as compared to controls.

Four modules were specifically activated in the inflammatory phase (module 14, 37, 39 and 43), one in the acute (module 32) and late fibrotic (module 20) phases each. Two further modules showed high differential activity in more than one phase indicating that the modules may represent cellular processes relevant to all stages of the development towards lung fibrosis (module 22 and 28).

Overall, eight relevant modules seem to be a reasonable number of modules with relevance to an AO, when comparing this result to other studies. An *in vivo* rat study of silica-induced pulmonary fibrosis appling RNA-Seq and a subsequent WGCNA found 17 modules, two of which were classified as relevant for pulmonary fibrosis (Lv et al., 2022). Another study investigated different stages of liver cirrhosis and considered seven modules to be relevant (Lu et al., 2023). The authors of the TXG mapper detected, in liver related datasets, 398 modules and grouped them into 8 clusters (the exact number of modules was not described) (Callegaro et al., 2021).

To increase the confidence in the specific relation of modules to the fibrotic mode of action and to specific subprocesses, the hub genes (i.e. genes that are particularly representative of the module) were examined.

The hub genes’ annotations to biological processes found in the literature corresponded very well with the histopathologically determined inflammatory as well as acute and late fibrotic processes in the bleomycin study. Exceptions include ZC3H18 and CL57BL6, the hub genes of modules 37 and 20, for which no information about related biological processes was found and only involvement in processes not related to fibrosis have been described in the literature. Interestingly, for the proposed potential biomarker modules not only the hub genes corresponded well with histopathologically determined phase. Instead, all of these modules included further genes that are known biomarkers of fibrosis. This finding supports the notion that the described modules should be used as composite biomarkers instead of proposing only e.g. hub genes of interesting modules as biomarkers, as has been done in biomedical studies such as (Tian et al., 2020; Wan et al., 2018; Yuan et al., 2021). In comparison to considering single genes as biomarkers, be it hub genes or genes with known relations to fibrotic processes as listed in DisGenet, the 8 modules described in this study may serve as much more robust biomarkers, because they build on expression patterns of dozens of genes, not only of single genes. In support of this theoretical argument, enrichment analysis results indicate that the modules reflect processes involved in inflammation and (fibrotic) remodelling of tissue.

The modules proposed in this study were detected without building on prior knowledge from sources such as pathway databases, which bear the possibility of bias towards already studied mechanisms. Eradicating this potential source of bias is a clear advantage. To our knowledge, a WGCNA analysis has not yet been performed on an *in vivo* data set for lung fibrosis covering the development of an adverse outcome over several phases, so modules can be assigned to the individual phases.

The proposed modules can be used for interpretation of mechanisms of action in applications such as read-across, where the mechanistic similarity of compounds needs to be characterized (Escher et al., 2019). Mechanistic similarity is difficult to assess based on single gene changes, as expression patterns of single genes are typically too variable (Escher et al., 2022b; Vrijenhoek et al., 2022). In contrast, pathway analysis or modules reduce the noise in these data and make it easier to detect compounds with similar mechanisms of action, as recently demonstrated in several case studies in the EUTOXRISK and RISK-HUNT3R projects (Drake et al., 2023; Escher et al., 2022a; Vrijenhoek et al., 2022).

The eight proposed biomarker modules were applied to an *in vitro* gene expression dataset for other known inducers of lung fibrosis (Drake et al., 2023) to explore their applicability domain (i.e., to which extent the modules reflect general responses to inducers of lung fibrosis). (Drake et al., 2023). reported a dose-dependent increase in the number of DEGs and DEGs’ fold change values, both in cells exposed to any of the three tested alpha-diketones and tunicamycin. The expression patterns induced by alpha-diketones have been found to be very similar by the authors, whereas both similarities and differences have been recognised in the expression pattern of tunicamycin. Interestingly, even though the total number of DEGs found for tunicamycin was comparable to those of the alpha-diketones, tunicamycin induced differential expression only in small fractions (<20%) of the genes within the proposed biomarker modules except for two inflammation related modules, where fractions reached or surpassed that level. This finding is expected, as tunicamycin is known to induce differential expression of genes involved in inflammation (Guo et al., 2017) but not in lung fibrosis. Further, this suggests that six of the eight modules represent specifically lung-fibrosis related processes.

One limitation of this case study is the small gene set (S1500+ panel) of the *in vitro* study, therefore a complete gene profile was not analysed and should be investigated in further case studies. Nevertheless, it could be shown that the fibrosis-inducing substances activate all identified modules, whereas the substance with different mode of action shows activity at most in the inflammatory phase.

The WGCNA analysis was based on mice exposed at a single dose level, so that dose-dependence could not be taken into account in generating the eight biomarker modules. Further dose groups would allow to compare low dose and high dose effects and focusing on genes that are regulated in a dose-dependent manner could minimise noise in the data.

As the *in vitro* dataset included only two to four concentrations, benchmark dose analysis could not be applied to it. Nevertheless, differential expression of genes and eigenvalues of the modules were investigated to characterize the type and trends in the concentration-responsiveness of the modules. The eigenescore (EG) analysis considered the absolute log2 foldchange values of all genes in a given module, regardless of whether they were differentially expressed. Therefore, after mapping a module to the gene expression, the EG serves as a measure of the overall activation of the module. Modules with a concentration-responsive EG (i.e., higher EG with increasing concentration) were considered activated with high reliablilty.

Considering both the number of DEGs as a fraction of measured genes in a given module and eigenvalues of the module has different advantages. One advantage of analysing the relative number of DEGs is that it is not biased when only a limited set of genes is measured in an experiment where the modules are applied as biomarkers. In contrast, eigenvalues can be biased by a reduced coverage of the modules. In turn, eigenvalues are based on more information about the activation of a module in a given experiment.

In the validation dataset both measures responded as expected. Dose-responsiveness of module 43, 32, 20, 22 and 28 eigenvalues is only visible for the fibrosis-inducing substances. The relative number of DEGs induced by the fibrosis-triggering substances is high for all modules whereas in tunicamycin treated cells it is only high for two modules that represent the inflammatory phase.

## Conclusion and outlook

The gene modules proposed as markers of lung fibrosis are based not only on the final fibrotic phase, but also on earlier events. More precisely, we can discern modules, which are particularly active at particular stages in the development towards lung fibrosis and others which are activated throughout the development. The modules themselves can serve as markers of the potential induction of lung fibrosis. Furthermore, they include genes, which have previously not been associated with lung fibrosis. In that sense, they are also a tool to generate new hypotheses regarding mechanisms underlying compound induced lung fibrosis. Results of hub gene characterization and mapping with information about known biomarkers from DisGenet confirm, that modules with high differential activity are indeed highly relevant for characterising fibrotic processes. Relating modules’ activity specifically to particular stages of the development of bleomycin-induced lung fibrosis, opened up for the possibility of mapping the modules to known AOPs and thus an opportunity to connect AOPs and transcriptomics data.

In conclusion, new biomarkers correlated with the development of compound-induced lung fibrosis were developed and applied in a proof-of-concept case study.

## Data Availability

Publicly available datasets were analyzed in this study. This data can be found here: https://www.ncbi.nlm.nih.gov/geo/query/acc.cgi?acc=GSE40151: GSE40151; https://www.ebi.ac.uk/biostudies/eu-toxrisk/studies?facet.eutoxrisk.project_part=cs8: S-TOXR1829, S-TOXR1814, S-TOXR1824, S-TOXR1825, S-TOXR1826, S-TOXR1827.
